# Maize *Carbohydrate partitioning defective1* impacts carbohydrate distribution, callose accumulation, and phloem function

**DOI:** 10.1093/jxb/ery203

**Published:** 2018-05-26

**Authors:** Benjamin T Julius, Thomas L Slewinski, R Frank Baker, Vered Tzin, Shaoqun Zhou, Saadia Bihmidine, Georg Jander, David M Braun

**Affiliations:** 1 Division of Biological Sciences, Interdisciplinary Plant Group, and Missouri Maize Center, University of Missouri-Columbia, Columbia, MO, USA; 2 Department of Biology, Pennsylvania State University, State College, PA, USA; 3 Boyce Thompson Institute, Ithaca, NY, USA

**Keywords:** Benzoxazinoid, callose, chlorosis, *Cpd1*, lignin, maize, phloem, starch, [^14^C]sucrose, sugars

## Abstract

Plants synthesize carbohydrates in photosynthetic tissues, with the majority of plants transporting sucrose to non-photosynthetic tissues to sustain growth and development. While the anatomical, biochemical, and physiological processes regulating sucrose long-distance transport are well characterized, little is known concerning the genes controlling whole-plant carbohydrate partitioning. To identify loci influencing carbon export from leaves, we screened mutagenized maize plants for phenotypes associated with reduced carbohydrate transport, including chlorosis and excessive starch and soluble sugars in leaves. *Carbohydrate partitioning defective1* (*Cpd1*) was identified as a semi-dominant mutant exhibiting these phenotypes. Phloem transport experiments suggested that the hyperaccumulation of starch and soluble sugars in the *Cpd1/+* mutant leaves was due to inhibited sucrose export. Interestingly, ectopic callose deposits were observed in the phloem of mutant leaves, and probably underlie the decreased transport. In addition to the carbohydrate hyperaccumulation phenotype, *Cpd1/+* mutants overaccumulate benzoxazinoid defense compounds and exhibit increased tolerance when attacked by aphids. However, double mutant studies between *Cpd1/+* and benzoxazinoid-less plants indicate that the ectopic callose and carbon hyperaccumulation are independent of benzoxazinoid production. Based on the formation of callose occlusions in the developing phloem, we hypothesize that the *cpd1* gene functions early in phloem development, thereby impacting whole-plant carbohydrate partitioning.

## Teaser

The maize *Carbohydrate partitioning defective1* mutant causes ectopic callose formation in sieve elements, which partially inhibits carbohydrate partitioning and is associated with hyperactive defense responses under insect pest exposure.

## Introduction

The transport of carbohydrates from photosynthetic source tissues (leaves) to non-photosynthetic sink tissues (e.g. roots, reproductive organs, and stem) is crucial for plant growth, development, and yield (Lalonde *et al.*, 2003; Slewinski and Braun, 2010*a*; Braun *et al.*, 2014; Yadav *et al.*, 2015; Julius *et al.*, 2017). In the majority of crop species, including maize (*Zea mays*), sucrose is the predominant (or sole) carbohydrate transported long distance for delivery to different sink tissues (Heyser *et al.*, 1978; Ohshima *et al.*, 1990). This process, termed whole-plant carbohydrate partitioning, occurs in the phloem tissue of the veins (van Bel, 2003; Ayre, 2011). Phloem tissue is composed of three cell types: phloem parenchyma (PP) cells, companion cells (CCs), and sieve elements (SEs); however, the SEs are enucleate and depend on neighboring CCs for metabolic support (van Bel and Knoblauch, 2000). Therefore, these two cell types function as a unit and are referred to as the CC/SE complex (Esau, 1977). There are few plasmodesmatal connections between the PP cells and the CC/SE complexes in maize leaves (Evert *et al.*, 1978). Therefore, sucrose must be exported across the plasma membrane into the cell wall space, or apoplasm, before being imported into the CCs (Slewinski *et al.*, 2009, 2010; Baker *et al.*, 2012, 2016; Braun, 2012; Chen *et al.*, 2012). Sucrose then moves cell to cell symplasmically through plasmodesmata (PDs) into the SEs, which are aligned end to end to form sieve tubes that act as a conduit for long-distance sucrose transport from source tissues to the sinks (Evert, 1982). The SE lumens are connected at the end walls by large arrays of PDs (i.e. sieve plates) that modulate the bulk flow of sieve tube sap by selective deposition or degradation of callose, a β-1,3-linked glycan, within the pores of the PDs (Chen and Kim, 2009; Xie *et al.*, 2011). Callose deposits are known to block cell to cell movement of solutes, hormones, and proteins, and therefore the control of callose deposition is a crucial mechanism of phloem sap transport, resource allocation, and plant development (van Bel, 2003; Chen and Kim, 2009; Guseman *et al.*, 2010; Barratt *et al.*, 2011; Vatén *et al.*, 2011; Xie *et al.*, 2011; Piršelová and Matušíková, 2013; Han *et al.*, 2014; Cui and Lee, 2016).

Plant pests and pathogens target the long-distance transport systems within veins to acquire resources and to spread to different tissues throughout the plant (Samuel, 1934; Carrington *et al.*, 1996; Oparka and Cruz, 2000; Bové and Garnier, 2003; Will and van Bel, 2006; Sun *et al.*, 2013). To counteract this attack, plants have evolved chemical defense responses not only to limit pathogen feeding and nutrient acquisition, but also to modulate the transport process to limit the spread of infectious agents from the site of entry (Aist, 1976; Iglesias and Meins, 2000; Hopkins *et al.*, 2009; Luna *et al.*, 2011). One such family of compounds is the benzoxazinoids, which are predominantly found in grasses, such as maize and wheat (*Triticum aestivum*) (Zúñiga *et al.*, 1983; Frey *et al.*, 2009; Ahmad *et al.*, 2011). In response to chemical signals associated with insect feeding or microbial attack, ectopic callose is deposited over the sieve plate pores to limit phloem transport (Iglesias and Meins, 2000; van Bel, 2003; [Bibr CIT0085][Bibr CIT0052]; Koh *et al.*, 2012).

Toward identifying genes that regulate carbohydrate allocation and ultimately influence crop productivity in maize, we screened mutagenized populations for mutants with visible phenotypes associated with carbohydrate hyperaccumulation in leaves, such as decreased plant growth, chlorotic leaves, and reduced fertility. Five maize mutants have been reported to express these phenotypes and found to function in carbohydrate partitioning: *sucrose export defective1* (*sxd1*), *sucrose transporter1* (*sut1*), *psychedelic* (*psc*), and the *tie-dyed1* (*tdy1*) and *tdy2* mutants (Russin *et al.*, 1996; Braun *et al.*, 2006; Baker and Braun, 2008; Braun and Slewinski, 2009; Slewinski *et al.*, 2009; Slewinski and Braun, 2010*b*; Baker *et al.*, 2013). Interestingly, the *sxd1* mutant exhibits ectopic callose deposits over the PDs between the bundle sheath (BS) and PP cells of leaf minor veins (Botha *et al.*, 2000). Occlusions in these PDs were suggested to inhibit symplasmic transport of sucrose, and thus sucrose export from the mature leaf. In contrast, *psc*, *sut1*, *tdy1*, or *tdy2* mutants have not been reported to exhibit ectopic callose deposition in leaf veins (Baker and Braun, 2008; Ma *et al.*, 2008; Slewinski *et al.*, 2010, 2012).

In addition to the above-mentioned maize mutants with defects in carbon export from leaves, we have identified many additional loci with similar overall phenotypes. Here, we characterize the maize mutant *Carbohydrate partitioning defective1* (*Cpd1*). Besides reduced plant growth, chlorotic leaves, and overaccumulation of non-structural carbohydrates (NSCs) in leaves, the mutant displays several additional, unique phenotypes that shed light on its function.

## Materials and methods

### Plant materials and growth conditions

To screen for putative *cpd* mutants, mutagenized maize populations were evaluated for families segregating small, slow-growing, chlorotic plants, and, in conducive genetic backgrounds, anthocyanins in the leaves. A confirmatory screen was performed on the identified mutant lines to test whether the mutants overaccumulated starch and, if so, they have been included in our *cpd* mutant collection. Over multiple years of screening, and with additional alleles donated from collaborators, we have identified nearly 100 mutations that give varying levels of the *cpd* suite of phenotypes. In this report, we characterize *Cpd1*, which was generated by Gerry Neuffer (University of Missouri) using ethyl methanesulfonate mutagenesis of maize pollen (Neuffer and Coe, 1978).

Maize plants used for the morphometrics, photosynthetic and gas exchange analyses, microscopy, and soluble sugar and starch quantifications were grown in the field at the University of Missouri South Farm Agricultural Experiment Station in Columbia, MO. *Cpd1/+* backcrossed into the inbred line B73 at least four times was used for all experiments. The *benzoxazinoneless1* (*bx1*) and *bx2* mutations, which have been previously described by Tzin *et al.* (2017), were also utilized. For the morphometric studies, two families segregating for the *Cpd1*/+ mutation in a 1:1 mutant:wild-type ratio were analyzed. Sample sizes for days to anthesis and days to silking measurements in the wild type and *Cpd1/+* mutants were *n*=40 and *n*=43; ear length measurements were *n*=39 and *n*=41; 100 kernel weight measurements were *n*=36 and *n*=37; kernel number measurements were *n*=17 and *n*=17; and plant height measurements were *n*=39 and *n*=44, respectively.

Plants used for the carboxyfluorescein diacetate (CFDA) and [^14^C]sucrose transport assays, innate benzoxazinoid measurements, and microscopy were grown in a greenhouse supplemented with high-pressure sodium lighting (1600 μmol m^−2^ s^−1^) under 16 h light and 8 h darkness. The plants were kept at a daytime and night-time temperature of 30 °C and 24 °C, respectively. Additionally, etiolated plants were grown for 13–15 d under 24 h dark conditions at 22 °C. Plants were PCR-genotyped to identify *Cpd1* homozygous mutants, heterozygotes, and homozygous wild-type individuals using the primer sequences Forward, 5'-ttgccaaaggtcagttagatgat-3'; and Reverse, 5'-aaattggctggtaactgacagaa-3'.

Maize plants used for the aphid and caterpillar assays and the post-aphid benzoxazinoid measurements were sown in plastic pots (~200 cm^3^) filled with moistened growth medium [produced by mixing 0.16 m^3^ Metro-Mix 360 (Scotts), 0.45 kg of finely ground lime, 0.45 kg of Peters Unimix (Scotts), 68 kg of Turface MVP (Profile Products), 23 kg of coarse quartz sand, and 0.018 m^3^ pasteurized field soil]. Plants were grown for 2 weeks in growth chambers under a 16 h light/8 h dark photoperiod and 180 μmol m^−2^ s^−1^ light at constant 23 °C and 60% humidity.

### Starch staining

Leaves were cleared and stained with iodine–potassium iodide as previously described (Baker and Braun, 2007).

### [^14^C]Sucrose and CF transport studies

Carboxyfluorescein (CF) and [^14^C]sucrose transport assays were conducted as previously described (Ma *et al.*, 2009; Slewinski *et al.*, 2009). For each experiment, three independent plants of each genotype, *Cpd1/+* and the wild type, were analyzed, and the experiments were repeated three times (*n*=9 of each genotype analyzed). For the *Cpd1/+* mutants, leaves that were fully chlorotic were abraded near the tip to apply the dye or radiotracer. In both experiments, leaves were harvested after 1 h of transport time, and cross-sectional images taken for the CF studies were located ~15 cm proximal to the application site, eliminating diffusion as a possible explanation for transport. Representative images of the results are shown.

### Photosynthesis and gas exchange measurements

Gas exchange, and photosynthetic rate and capacity measurements were taken on mature source leaves between 9 am and 12 noon using a portable infrared gas exchange system (LI-6400XT, LI-COR Inc., Lincoln, NE, USA) as described (Bihmidine *et al.*, 2015; Huang *et al.*, 2009). Chlorophyll content was measured using a Minolta SPAD-502 meter (Spectrum Technologies, Plainfield, IL, USA) (Ma *et al.*, 2008). Net photosynthesis (*A*_net_, μmol CO_2_ m^−2^ s^−1^) and stomatal conductance (*g*_s_, mol H_2_O m^−2^ s^−1^) rates were measured at a photon flux density of 2000 μmol m^−2^ s^−1^ and ambient CO_2_ concentration of 400 μmol mol^−1^. The maximum photochemical efficiency of PSII (*F*_v_/*F*_m_) was determined on dark-adapted leaves using a leaf fluorometer attached to the LI-6400XT infrared gas analyzer at an ambient CO_2_ concentration of 400 μmol mol^−1^. For the *Cpd1/+* mutant leaves, the regions analyzed for the *F*_v_/*F*_m_ experiment contained a mixture of both chlorotic margins expressing anthocyanins and greener tissue closer to the mid-rib. Five biological samples were measured for both the wild type and *Cpd1*/+ mutants.

### Light and fluorescence microscopy

Microscopy images were taken using a Nikon Eclipse 80i epifluorescent microscope equipped with a 100 W mercury bulb and a DXM1200F camera, except for the aniline blue staining of the immature leaf gradient and the etiolated seedling images in which LED lights (Lumencore Sola SMII light engine) replaced the 100 W mercury bulb. Mature leaf tissue free-hand cross-sections were generated and imaged as previously described (Baker *et al.*, 2016). Callose was visualized under UV excitation by staining mature leaf sections with 0.005% (w/v) aniline blue in 0.15 M potassium phosphate buffer (pH 8.2) (Ruzin, 1999). Lignin was visualized by staining leaf sections with Maule stain (0.5% KMnO_4_) for 10 min. The leaf sections were subsequently rinsed in water, de-stained in 10% HCl for 5 min, and again rinsed in water before mounting in concentrated NH_4_OH and imaging them (Chapple *et al.*, 1992).

### Soluble sugar and starch quantification

Mature source leaf tissue was harvested from field-grown plants and immediately placed in liquid nitrogen before being stored at –80 °C until measurement. End of day (EOD) samples were harvested at 16.30 h, and end of the night (EON) samples were harvested at 05.30 h. Five biological samples were measured for each genotype and time point. Soluble sugar and starch samples were extracted according to Leach and Braun (2016). Samples were quantified using high-performance anion exchange (HPAE) chromatography against known standards according to Leach *et al.* (2017).

### Aphid bioassays

Corn leaf aphids (*Rhopalosiphum maidis*) were reared on the maize line B73 as previously described (Meihls *et al.*, 2013). For aphid bioassays, 10 adult aphids were confined on 2-week-old seedling plants using micro-perforated polypropylene bags. Five days after the infestation, the aphid progeny were counted and the number of nymphs produced per adult per day was calculated. Sample sizes for wild-type and *Cpd1/*+ mutant plants were *n*=17 and *n*=9, respectively.

### Caterpillar bioassays

Eggs of three lepidopteran species, *Spodoptera exigua* (beet armyworm), *Spodoptera eridania* (southern armyworm), and *Spodoptera frugiperda* (fall armyworm), were purchased from Benzon Research Inc. (Carlisle, PA, USA). *Spodopter*a eggs were hatched in a 29 °C incubator (~48 h). First-instar larvae (one larva per plant) were confined on 2-week-old seedling plants using micro-perforated polypropylene bags. Ten days after infestation, the larvae were collected, lyophilized, and weighed. Sample sizes for wild-type and *Cpd1*/+ mutant plants were, for beet armyworm *n*=4 and *n*=3; for southern armyworm, *n*=5 and n=5; and for fall armyworm, *n*=10 and *n*=5, respectively.

### Benzoxazinoid measurements

For measurement of leaf benzoxazinoid content post-aphid treatment, maize leaf tips (~5 cm) of the third and fourth leaves were independently collected from plants that had been fed upon by aphids for 5 d. Similar tissue samples were taken from individuals not exposed to aphids for innate benzoxazinoid measurements. Metabolite extraction and quantification with LC-MS analysis were performed as previously described (Handrick *et al.*, 2016). Sample sizes for post-aphid treatment for wild-type and *Cpd1/*+ mutant plants were *n*=17 and *n*=9, respectively. Sample sizes for innate benzoxazinoid measurements for wild-type and *Cpd1*/+ mutant plants were *n*=8 and *n*=7, respectively.

## Results

### The *Cpd1* mutation results in shorter plants with pigmented leaves

**Fig. 1.**
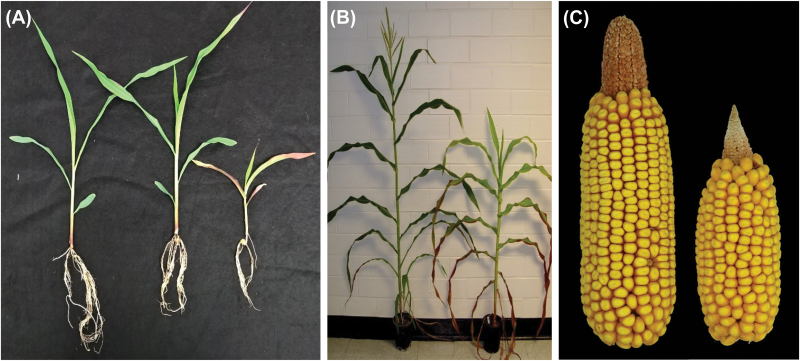
*Cpd1* mutants have diminished plant stature and reduced ear size relative to their wild-type siblings. (A) Two-week-old sibling plants: wild-type on the left, heterozygous *Cpd1*/+ mutant in the middle, and homozygous *Cpd1* mutant on the right. (B) A wild-type plant (left) at maturity and its *Cpd1*/+ mutant sibling (right) at the same age exhibiting diminished stature and delayed development. (C) A wild-type ear (left) and *Cpd1*/+ mutant ear (right) displaying the reduced ear size of the mutant.

The Mendelian segregation ratio of the *Cpd1* mutant indicates that it is conditioned by a semi-dominant mutation (Fig. 1A; Supplementary Table S1 at *JXB* online). The homozygous mutant exhibits delayed growth in the shoot and root, resulting in a stunted appearance (Fig. 1A). Additionally, the shoot exhibits a highly variable continuum of light green to chlorotic leaves, which rapidly progresses to necrosis, initiating from the tip and progressing basipetally. Ultimately, the homozygous mutant is seedling lethal and survives only 2 weeks after planting. Heterozygote mutants (*Cpd1*/+) grow to maturity, but exhibit regions of chlorosis and anthocyanin accumulation in the source leaves as early as 2 weeks after germination. *Cpd1/+* mutants also display decreased plant stature and shorter ear length compared with wild-type siblings (Fig. 1A–C). Additionally, *Cpd1/+* mutant plants are developmentally delayed, requiring more time to reach anthesis and silking; however, the number of kernels per ear is not significantly different from that of the wild type, although kernel weight was significantly reduced in the mutant (Table 1). For all of our analyses, the *Cpd1* mutation was backcrossed four or more times to the B73 inbred line, thereby greatly reducing the likelihood that other mutations are segregating in the background.

**Table 1.** *Cpd1/+* mutants show reduced height and yield, and delayed flowering

	*Cpd1*/+ (mean ±SE)	Wild type (mean ±SE)
Plant height (cm)	214.89 ± 1.60*	223.03 ± 1.39
Days to anthesis	61.00 ± 0.24*	57.63 ± 0.20
Days to silking	62.25 ± 0.23*	58.72 ± 0.19
Ear length (cm)	121.83 ± 2.42*	148.89 ± 1.52
Weight of 100 kernels (g)	22.99 ± 0.36*	26.00 ± 0.26
Kernel number per ear	330.71 ± 20.23	348.82 ± 14.09

An asterisk signifies significantly different means between *Cpd1*/+ mutant and wild-type plants at *P*≤0.05, using a two-tailed Student’s *t*-test.

**Fig. 2.**
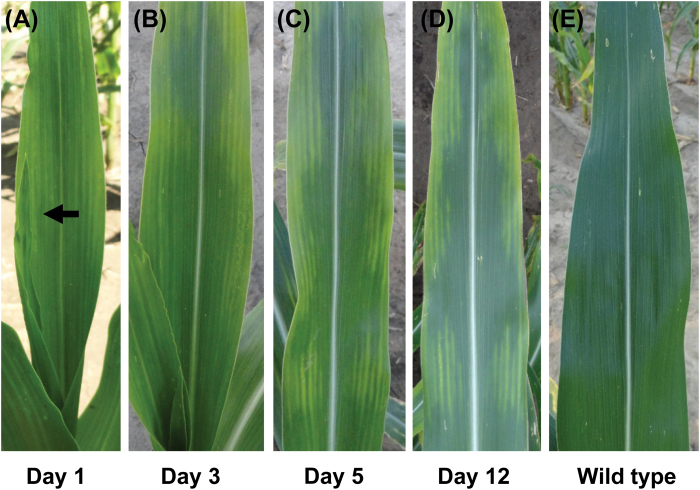
Developmental time course of the *Cpd1/+* mutant leaf chlorosis phenotype. The leftmost leaf image (A) was photographed beginning 5 d post-emergence of the tip of the leaf from the whorl. Day 1 was the first day that the border between emergent pale-chlorotic (top) and normal regions (bottom) was evident (arrow), and was also the day that the region containing this border had emerged. The chlorotic sectoring pattern became progressively more visible, but remained unaltered (did not spread) over the life span of the leaf (B–D). Minor differences in the size of sectors between images are due to further emergence from the whorl and slight daily differences (e.g. angle) in the photographing of the leaf. A wild-type leaf is shown on the far right for comparison (E).

To characterize when the defect first appeared in *Cpd1/+* mutant leaves, we examined mutant leaves of field-grown plants from their initial emergence from the whorl to monitor phenotypic progression. The *Cpd1/+* mutant phenotype first manifests in the tip of leaves as slightly chlorotic regions as the leaves emerge from the whorl, with new chlorotic regions continuing to appear in a basipetal fashion as leaf emergence continues (Fig. 2). While the surrounding leaf tissue appears to undergo normal greening, the chlorotic regions progressively increase in severity over time, and anthocyanin accumulation typically occurs as chlorosis increases. Once formed, the chlorotic regions do not appear to increase in size and do not revert to dark green tissue.

### *Cpd1/+* mutant leaves accumulate excess starch and sugars

**Fig. 3.**
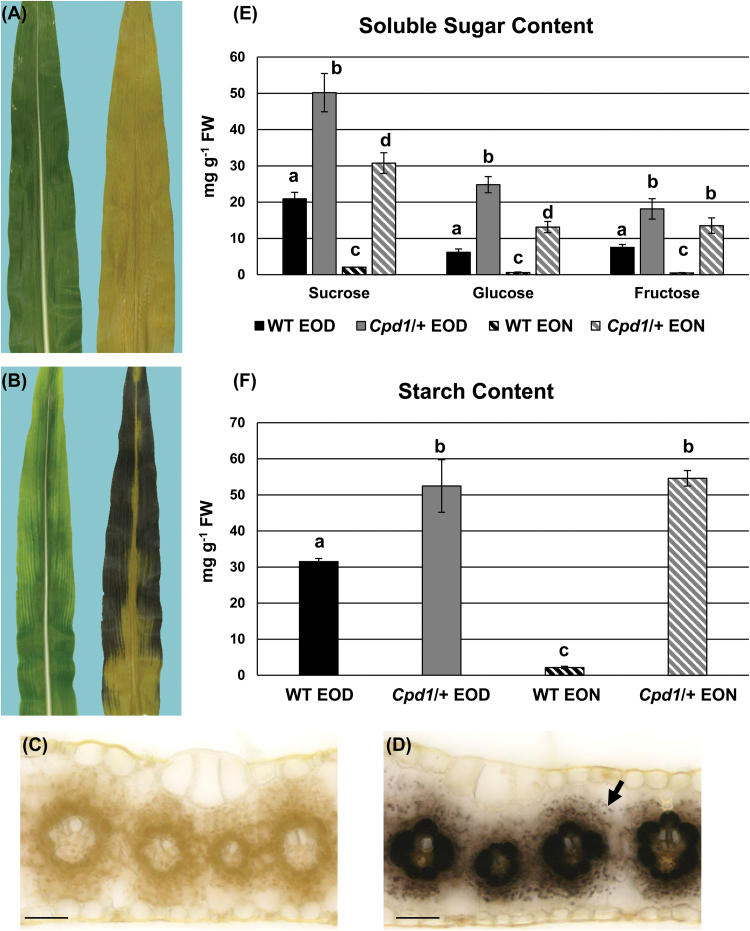
Non-structural carbohydrate quantification and starch staining of *Cpd1*/+ mutant and wild-type sibling leaves. (A and B) Wild-type (WT) (A) and *Cpd1*/+ mutant (B) mature leaf photographs before (left) and after starch staining (right). (C and D) Cross-sections of starch-stained leaves in WT (C) and *Cpd1*/+ mutant mature leaves (D) at end of the night (EON). The arrow indicates starch accumulation in a M cell plastid. Scale bar=50 µm. (E and F) Sucrose, glucose, and fructose (E), and starch (F) content in WT and *Cpd1*/+ mutant sibling leaves at end of the day (EOD) and EON time points. Black bars=WT EOD; gray bars=*Cpd1/+* mutant EOD; black and white striped bars=WT EON; gray and white striped bars=*Cpd1/+* mutant EON samples. Values are means ±SE, and significantly different values at *P*≤0.05 were determined using a two-tailed Student’s *t*-test, and are indicated with different letters.

Due to the similarity of the *Cpd1/+* mutant phenotype to *sxd1*, *sut1*, *psc*, and the *tdy* mutants, which all hyperaccumulate starch and sugars in their leaves, *Cpd1*/+ mutant and wild-type leaves were analyzed with potassium iodine staining to visualize starch accumulation. In this assay, regions of the leaves containing abundant starch appear black/brown, whereas those with little starch appear a pale tan color. No starch accumulation was detected in the wild-type leaves collected near dawn, indicative that the transitory stored starch was depleted over the course of the night due to remobilization (Fig. 3A). However, the *Cpd1/+* mutant leaves displayed intense starch accumulation in the chlorotic regions (Fig. 3B). To extend these results, free-hand cross-sections of these stained leaves were microscopically analyzed to determine in which cells the starch accumulated. In wild-type plants, no starch granules were present in the chloroplasts of BS and mesophyll (M) cells, consistent with the remobilization of starch over the course of the night (Fig. 3C) (Rhoades and Carvalho, 1944). However, in *Cpd1/+* mutants, both BS and M cells contained abundant starch, with the BS cells showing substantial staining (Fig. 3D). These results demonstrate that the mutant leaves hyperaccumulate starch, suggesting that *Cpd1/+* mutants are severely limited in the ability to remobilize stored carbon.

Given the elevated starch levels in *Cpd1/+* mutant leaves, we quantitatively assessed the levels of NSCs in mutant and wild-type leaves. Tissue samples were harvested at the EOD and EON from mature source leaves of field-grown plants when the mutants were beginning to display anthocyanin accumulation at the edge of the blade. Mutant leaf tissues contained ~2- to 4-fold the amount of soluble sugars and ~2-fold the amount of starch compared with their wild-type siblings in the EOD samples (Fig. 3E, F). However, the *Cpd1*/+ mutants contained ~14- to 28-fold the amount of soluble sugars and ~25-fold greater starch levels than their wild-type siblings in the EON samples. This large increase in soluble sugar and starch levels in the *Cpd1*/+ mutant leaves further suggests that the mutant is unable to export sucrose effectively from the source leaves.

Sugars can act as potent signals to down-regulate photosynthesis (Sheen, 1990, 1994; Goldschmidt and Huber, 1992; Krapp and Stitt, 1995; Koch, 1996; Jeannette *et al.*, 2000; Rolland *et al.*, 2006; Ruan, 2014). Therefore, we anticipated that the marked excess of sucrose and glucose in the mutant leaves would serve as sugar signals to repress photosynthesis. To test this idea, leaf photosynthetic capacity and gas exchange rates were measured for both mutant and wild-type plants. In comparison with the wild type, the chlorotic regions of *Cpd1*/+ mutant plants displayed a 50% decrease in leaf chlorophyll concentration, a 75% decrease in photosynthetic rate, and a 10% decrease in the maximum quantum efficiency of PSII photochemistry (Supplementary Fig. S1A–C). The *Cpd1*/+ mutant also showed a 68% decrease in stomatal conductance (Supplementary Fig. S1D). However, there was no difference between wild-type and mutant plants in the number of stomata on either leaf epidermal surface, indicating that the decreased gas exchange was not due to fewer stomata in the mutant leaves (data not shown). In summary, the data are consistent with the high sugar levels leading to feedback inhibition of photosynthesis and thus substantially decreased photosynthetic capacity in the mutants as compared with wild-type plants.

### Sucrose transport is inhibited in *Cpd1/+* mutant leaves

**Fig. 4.**
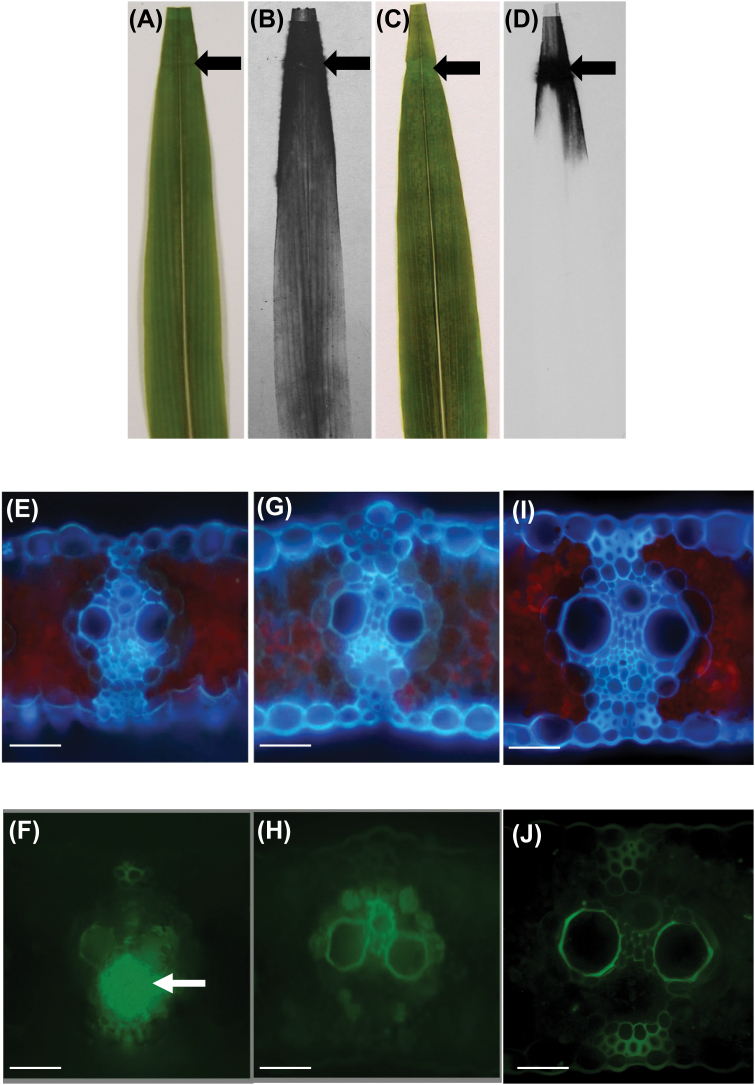
*Cpd1/+* mutant leaves exhibit decreased sucrose and carboxyfluorescein (CF) phloem transport. (A–D) ^14^C-Labeled sucrose transport in wild-type (A, B) and *Cpd1*/+ mutant (C, D) sibling leaves. The black arrow represents the abraded site on the leaf where the [^14^C]sucrose was applied. (A, C) Leaf photographs. (B, D) Autoradiograms of ^14^C signal. (E–J) Cross-sections of a lateral vein in wild-type (E, F, I, J) and *Cpd1*/+ mutant (G, H) sibling leaves with (F, H) and without (J) CF application examined under UV (E, G, I) or blue light (F, H, J). CF is abundant in the phloem of the wild-type vein (F, arrow), but not detected in the phloem of the *Cpd1*/+ mutant vein (H). Scale bar=50 µm.

To test directly whether phloem transport of sucrose is altered in the mutant, ^14^C-labeled sucrose was applied to an abraded region at the tip of a mature source leaf of an intact plant to observe its transport in both wild-type and *Cpd1*/+ mutant leaves. As expected, in the wild-type leaf, the [^14^C]sucrose was readily transported from the tip to the base of the leaf (Fig. 4A, B). However, in the *Cpd1*/+ mutant leaf, little to no transport of the [^14^C]sucrose occurred, with the majority of the label remaining at the application site (Fig. 4C, D).

To confirm these results, a phloem-mobile tracer, CFDA, was similarly applied to mature source leaves of both wild-type and *Cpd1/+* mutant plants. CFDA is cell permeable and, once inside the cell, is cleaved to form CF. CF is a fluorescent, charged molecule unable to move across cell membranes, and is transported through sieve tubes in a similar manner to sucrose (Grignon *et al.*, 1989; Ma *et al.*, 2009; Bihmidine *et al.*, 2015; Baker *et al.*, 2016). Leaf cross-sections were taken 15 cm proximal from the CFDA application site to determine if CF was transported through the phloem. In agreement with the [^14^C]sucrose transport data, CF was not present in the phloem of *Cpd1*/+ mutant leaf veins (Fig. 4G, H). These samples appeared similar to negative control sections to which no CF was applied and which were used to visualize tissue autofluorescence (Fig. 4I, J). However, CF fluorescence was clearly seen in the wild-type phloem (Fig. 4E, F). Thus, the *Cpd1/+* mutant is unable to transport sucrose and CF effectively through the phloem of mature source leaves, consistent with the hypothesis that sucrose export is partially inhibited. Furthermore, this transport failure could explain the hyperaccumulation of NSCs, leaf chlorosis, and reduction in photosynthetic performance in the mutant leaves.

### *Cpd1/+* mutants exhibit ectopic callose and lignin deposition in the phloem

**Fig. 5.**
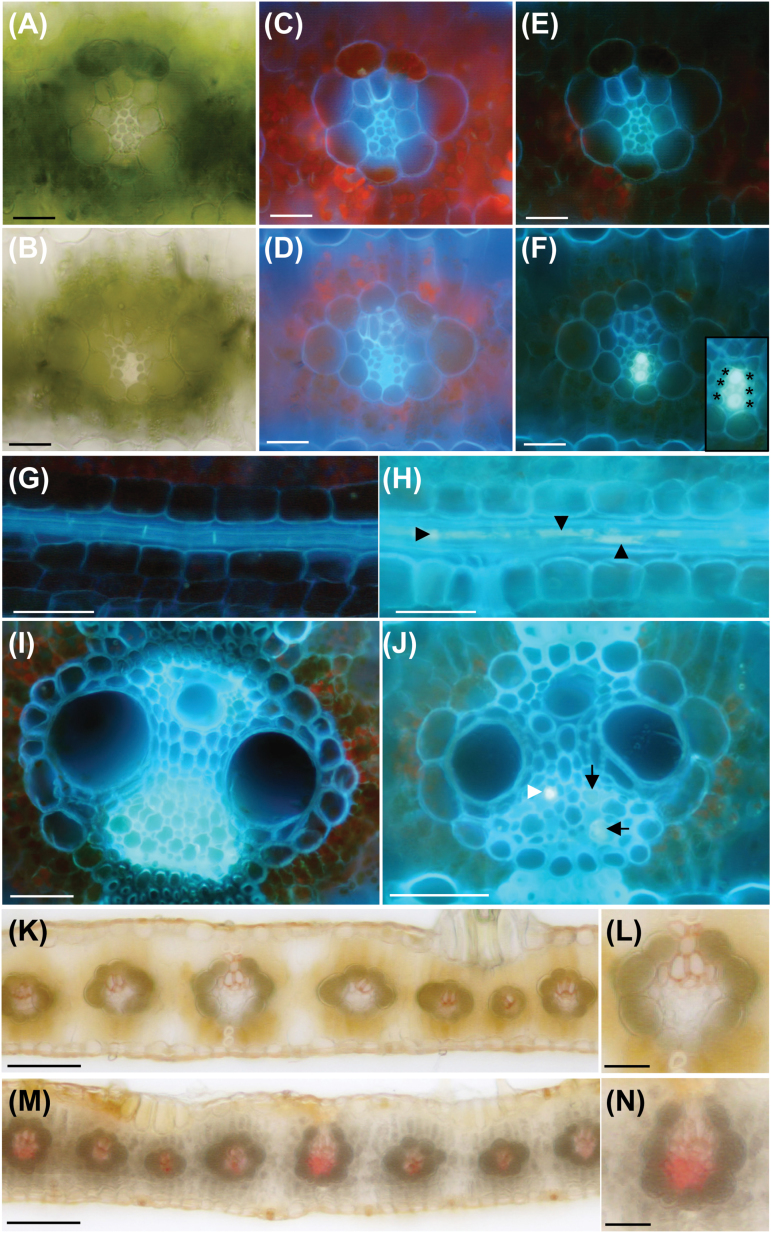
The phloem of *Cpd1*/+ mutant leaves contains ectopic callose and lignin deposition. (A, C, E, G, I, K, and L) Images of wild-type leaf sections; (B, D, F, H, J, M, and N) images of *Cpd1/+* mutant leaf sections. (A, B, K–N) Bright-field images; (C and D) UV autofluorescence, (E–J) aniline blue staining of callose under UV illumination. Callose deposition in cross-sections of *Cpd1/+* mutant minor (B, D, F) and lateral veins (J) observed under bright-field (B) and UV light (without and with aniline blue staining, D and F). The inset in (F) shows a close-up of the phloem region containing ectopic callose. Asterisks signify CCs (inset F). Paradermal longitudinal sections of wild-type (G) and *Cpd1/+* mutant (H) veins show that callose deposits along the length of the sieve tube are only observed in the mutant (arrowheads). In addition to callose staining by aniline blue (white arrowhead), non-aniline-blue-positive staining material can also be observed in the mutant *Cpd1*/+ vein (black arrows in J). Ectopic lignin deposition in the *Cpd1/+* mutant phloem was revealed by Maule staining in both minor and lateral veins in chlorotic tissue (M and N). Lignin was only observed in the xylem cell walls of wild-type tissue (K and L). Scale bars in A–F=25 µm; in G, H, K, M=100 µm; in I, J, L, N=50 µm.

Wild-type and *Cpd1*/+ mutant leaves were examined by light microscopy to determine whether anatomical differences could account for the inhibited phloem transport. No differences were observed in vein number or size, cell number or size, or overall leaf architecture in the mutants. Intriguingly, examination of cross-sections from mature *Cpd1/+* mutant chlorotic leaves beginning to accumulate anthocyanin revealed visible blockages in the phloem under both bright-field and UV illumination (Fig. 5B, D). No such blockages were observed in wild-type leaves sampled from the equivalent region (Fig. 5A, C). Histochemical analyses were performed on the leaf samples to determine the chemical nature of the blockages. Staining of the *Cpd1/+* mutant leaf sections with aniline blue, a dye that binds callose and fluoresces blue–white under UV light (Ruzin, 1999), showed greatly increased callose deposition in the phloem tissue in both the lateral and minor veins (Fig. 5F, H, J). These deposits were absent from the phloem tissue of wild-type samples (Fig. 5E, G, I). Based on the cell positions and sizes, these deposits appeared to be present in the SEs, but not the CCs or PP cells, although we cannot rule out the possibility that these other cell types also accumulate ectopic callose (Fig. 5F). Interestingly, in most veins analyzed, both callose-occluded and unoccluded sieve tubes were found (Fig. 5F, J). Further examination of the *Cpd1*/+ mutant leaves with Maule reagent, which stains lignin red, revealed ectopic lignin in the cell walls of the phloem tissue in regions with severe chlorosis and anthocyanin accumulation (Fig. 5M, N). Lignin was never observed in the phloem of the wild-type veins (Fig. 5K, L).

**Fig. 6.**
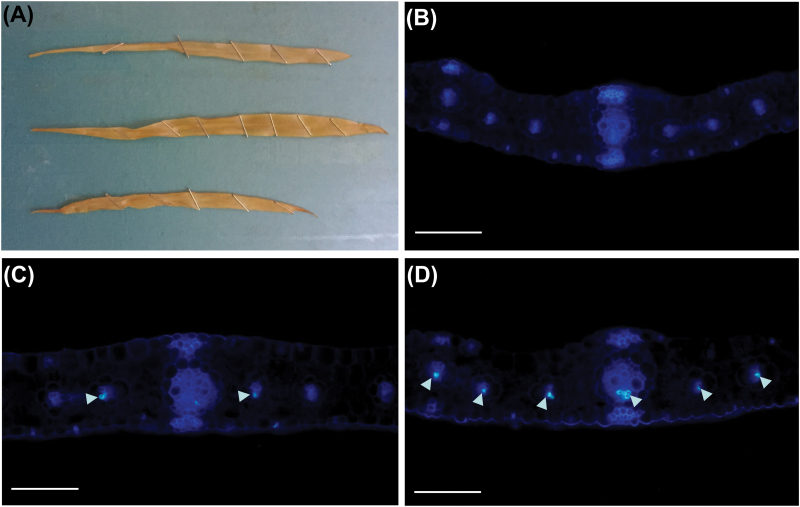
Ectopic callose is present in *Cpd1* mutant veins prior to starch accumulation. (A) Starch-stained second leaves of etiolated wild-type (top), *Cpd1*/+ heterozygote (middle), and *Cpd1*/*Cpd1* homozygous mutant (bottom) siblings. Staples were used for structural support as the leaves were fragile. (B–D) Aniline blue-stained cross-sections of etiolated juvenile leaf tissue from wild-type (B), *Cpd1*/+ heterozygote (C), and *Cpd1*/*Cpd1* homozygous mutant plants (D). Arrowheads indicate excess callose deposits. Scale bars=100 µm.

The above results afford a possible explanation for the hyperaccumulation of NSCs in *Cpd1/+* mutant leaves; that is, the ectopic callose in some sieve tubes impairs phloem transport and reduces sucrose export, resulting in sugar and starch build-up in mutant leaves. However, an alternative possibility is that NSC accumulation or exposure to light results in the callose deposition. To distinguish between these hypotheses, we analyzed etiolated *Cpd1*/*Cpd1* homozygotes, *Cpd1*/+ heterozygotes, and wild-type seedlings resulting from the self-fertilization of a heterozygote plant, since dark-grown seedlings are not expected to have high levels of NSCs in leaves. Fully expanded second leaves from etiolated seedlings were collected and starch stained. As predicted, none of the mutant or wild-type seedlings accumulated any detectable starch (Fig. 6A). Leaf samples were then sectioned and stained with aniline blue to determine whether callose deposits were present in the phloem tissue independent of starch hyperaccumulation. As expected, the wild-type plants did not have callose deposits in their phloem tissue (Fig. 6B). However, both the heterozygous and homozygous *Cpd1* mutant plants exhibited callose deposits in the lateral and minor veins, with the homozygous mutants having more numerous and severe deposits than heterozygous plants (Fig. 6C, D). These results support the hypothesis that the callose deposits inhibit phloem transport in the *Cpd1* mutant and precede the NSC accumulation in leaves and the other phenotypes.

### Callose deposition in *Cpd1/+* mutant plants occurs early in phloem development

**Fig. 7.**
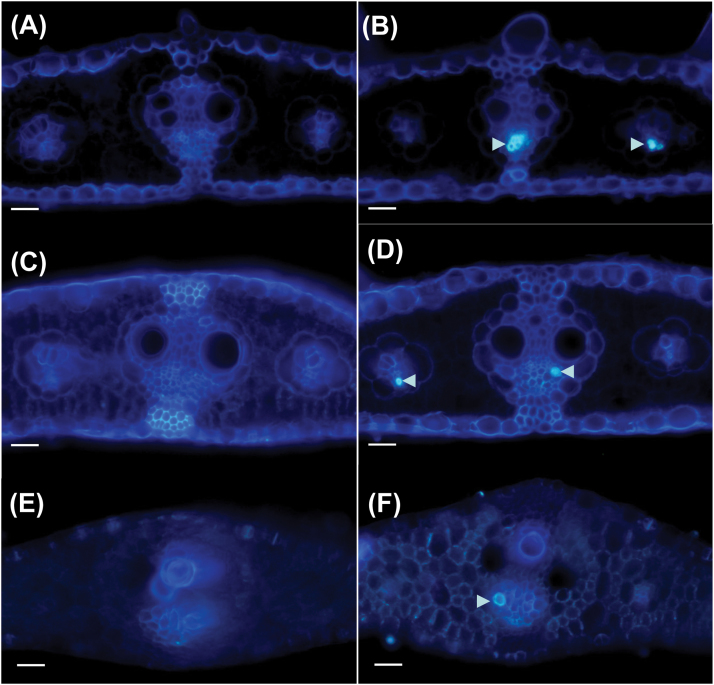
Cross-sections of expanding immature wild-type and *Cpd1*/+ mutant leaves stained with aniline blue and imaged under UV light. (A, C, and E) are images from different regions (tip, middle, and base, respectively) of the same wild-type leaf, and (B, D, and F) are images from these three regions from the same *Cpd1/+* mutant leaf. (A and B) Cross-sections from the tip of emerging immature leaves, which were becoming exposed to sunlight. (C and D) Cross-sections from the middle region of the immature leaves, which were still embedded within the whorl. (E and F) Cross-sections from the base of the immature leaves, which were embedded within the whorl. Arrowheads indicate excess callose deposits. Scale bars=50 µm.

Callose deposition and degradation are crucial steps in the development of mature SEs and their sieve plates (Esau *et al.*, 1962; Northcote and Wooding, 1966). Therefore, we hypothesized that misregulation of this process during vein development could result in the ectopic callose seen in the *Cpd1* mutant plants. To determine the approximate developmental time at which the excess callose forms in *Cpd1/+* mutant veins, immature leaves were harvested when their tips were just emerging from the whorl and were green, but their middle and bases were still within the whorl and pale yellow-green or yellow-white, respectively. Aniline blue staining revealed that the tips of the *Cpd1/+* mutant leaves contained high levels of callose in most minor veins and lateral veins, with no callose detected in corresponding wild-type samples (Fig. 7A, B). Similar results were seen in sections taken from the middle of the leaves (Fig. 7C, D). Interestingly, in cross-sections taken from the leaf base, very few callose deposits were present in the *Cpd1/+* mutant veins, and the majority were located in the lateral veins, as minor veins had just initiated development at this stage (Fig. 7E, F). At this point in leaf vein development, the metaphloem in the lateral vein has recently formed after the collapse of the protophloem (Evert *et al.*, 1996). No ectopic lignin was observed in wild-type or *Cpd1/*+ immature leaves using Maule reagent (data not shown). These results indicate that the callose deposits are occurring during the maturation of the phloem SEs.

### *Cpd1/+* mutants exhibit biotic stress responses

**Fig. 8.**
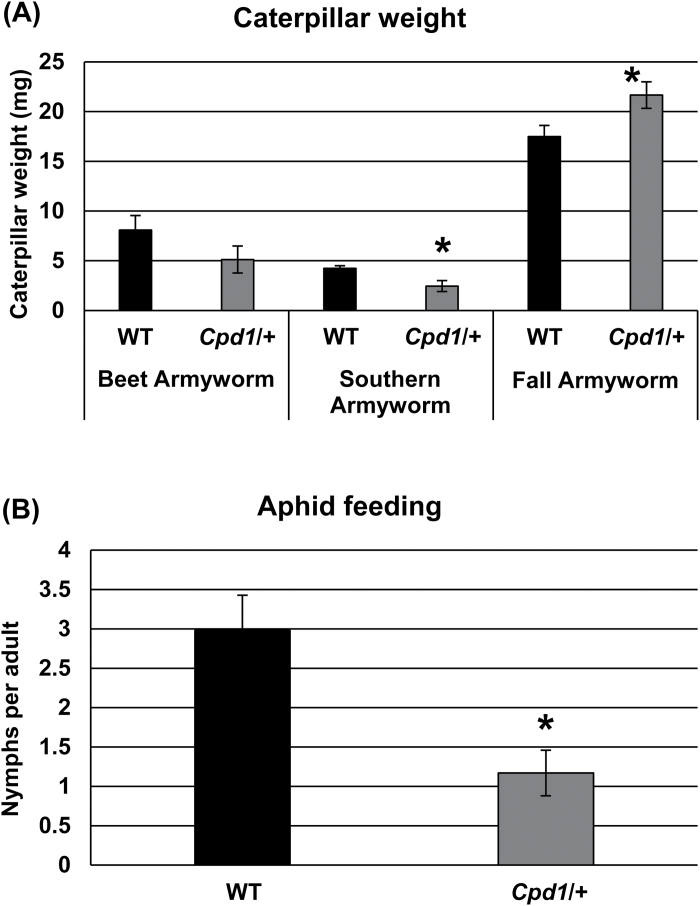
Chewing and non-chewing pest resistance in *Cpd1*/+ mutant plants compared with wild-type (WT) siblings. (A) Caterpillar weight after 10 d of feeding on *Cpd1*/+ mutant (gray bars) and WT (black bars) siblings. (B) Number of aphid progeny produced per adult aphid after 5 d feeding on *Cpd1*/+ mutant (gray bars) and wild-type (black bars) plants. Values are means ±SE, and an asterisk indicates significantly different means at *P*≤0.05, using a two-tailed Student’s *t*-test.

Callose deposition is often a defense response to pest or pathogen attack to limit nutrient loss. Therefore, we initially hypothesized that the callose deposition in the *Cpd1/+* mutant might be due to activation of defense response pathways. To test this hypothesis, caterpillar-feeding experiments were performed to determine whether *Cpd1/+* mutant plants exhibited enhanced defense against insects. Three species of Lepidoptera, the generalists beet armyworm (*Spodoptera exigua*) and southern armyworm (*Spodoptera eridania*), and the monocot-feeding specialist fall armyworm (*Spodoptera frugiperda*) were separately placed on wild-type and *Cpd1*/+ mutant 2-week-old seedlings and allowed to feed for 10 d. Interestingly, the fall armyworms that fed on the *Cpd1*/+ plants were significantly larger than those that fed on the wild-type plants at the end of the 10 d feeding period (Fig. 8A). The opposite effect was observed for the southern armyworm, as the insects feeding on the wild-type plants gained more weight (Fig. 8A). This trend also held for the beet armyworm. However, the average caterpillar weights of the two treatments were not significantly different for the beet armyworm (Fig. 8A).

Additionally, an aphid feeding experiment was conducted to test whether the *Cpd1*/+ mutant showed increased resistance against piercing/sucking pests, such as corn leaf aphids (*Rhopalosiphum maidis*), which are specialized for feeding on grasses. Adult aphids were placed on 2-week-old wild-type or *Cpd1*/+ mutant plants, and after 5 d their progeny were counted. Those feeding on the wild-type plants produced ~2-fold more nymphs per adult per day compared with those placed on *Cpd1/+* mutant plants (Fig. 8B). These data indicate that aphid fecundity was reduced on *Cpd1/+* mutant plants.

**Fig. 9.**
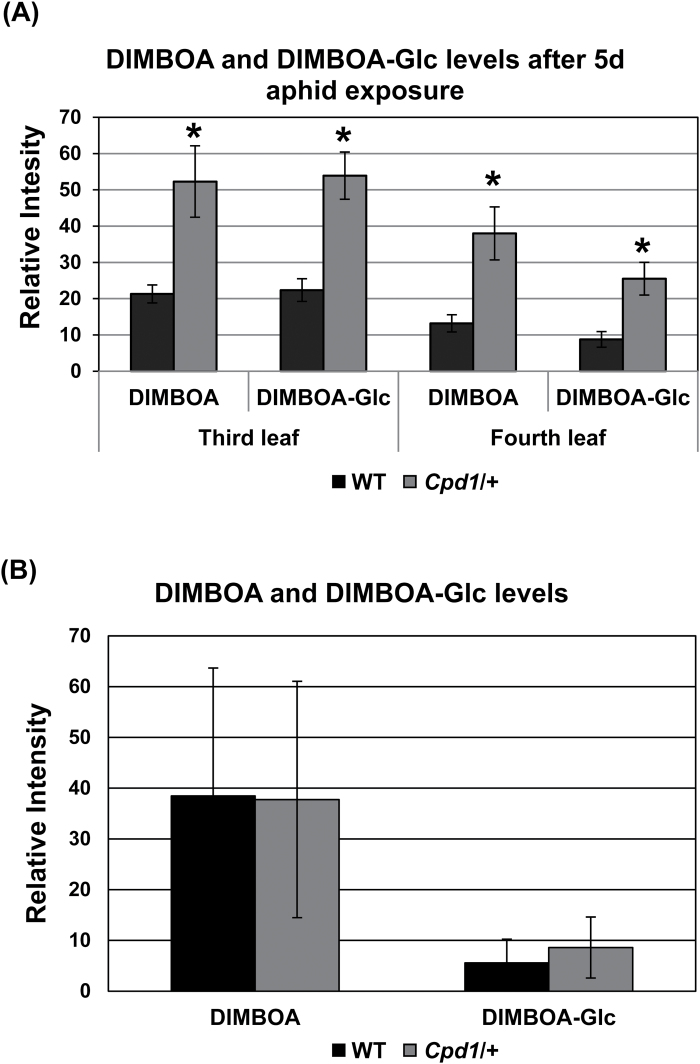
Benzoxazinoid (BX) levels in juvenile wild-type (WT) and *Cpd1/+* mutant leaves after *R. maidis* treatment (A) and plants not exposed to *R. maidis* (B). DIMBOA and DIMBOA-Glc levels were measured in the third and fourth leaves of the WT (black bars) and the *Cpd1/+* mutant (gray bars). Values are means ±SE, and an asterisk indicates significantly different means at *P*≤0.05, using a two-tailed Student’s *t*-test.

Callose deposition in maize leaves has been reported to be triggered by increased levels of 2,4-dihydroxy-7-methoxy-1,4-benzoxazin-3-one (DIMBOA), a member of the benzoxazinoid defense metabolite family, in the leaf apoplasm (Ahmad *et al.*, 2011). To determine if the above results are due to increased defense metabolite levels, the wild-type and *Cpd1/+* mutant leaves from the aphid feeding experiment were analyzed to determine whether the *Cpd1*/+ mutant leaves contained elevated DIMBOA levels. Indeed, we found that the *Cpd1/+* mutant plants contained ~2- to 3-fold higher levels of both DIMBOA and glucosylated-DIMBOA (DIMBOA-Glc) (Fig. 9A). As there may be some degradation of DIMBOA-Glc to DIMBOA during the extraction process, the reported DIMBOA abundance may be higher than what is actually in the leaves. These results are in agreement with the hypothesis that increased DIMBOA levels trigger callose deposition in the phloem tissue, resulting in the decreased carbohydrate transport and the mutant phenotype. Thus, the ectopic callose in *Cpd1/+* mutant leaves is associated with a poorer ability of the aphids to reproduce and with decreased growth of caterpillar herbivores, with the exception of the fall armyworm. This was probably due to this species’ ability to inactivate grass-specific metabolites, such as benzoxazinoids, in their gut (Glauser *et al.*, 2011; Maag *et al.*, 2014), which allowed the caterpillar to feed on the excess carbohydrates in the mutant leaves.

### DIMBOA-deficient mutants fail to rescue the *Cpd1/+* mutant phenotype

**Fig. 10.**
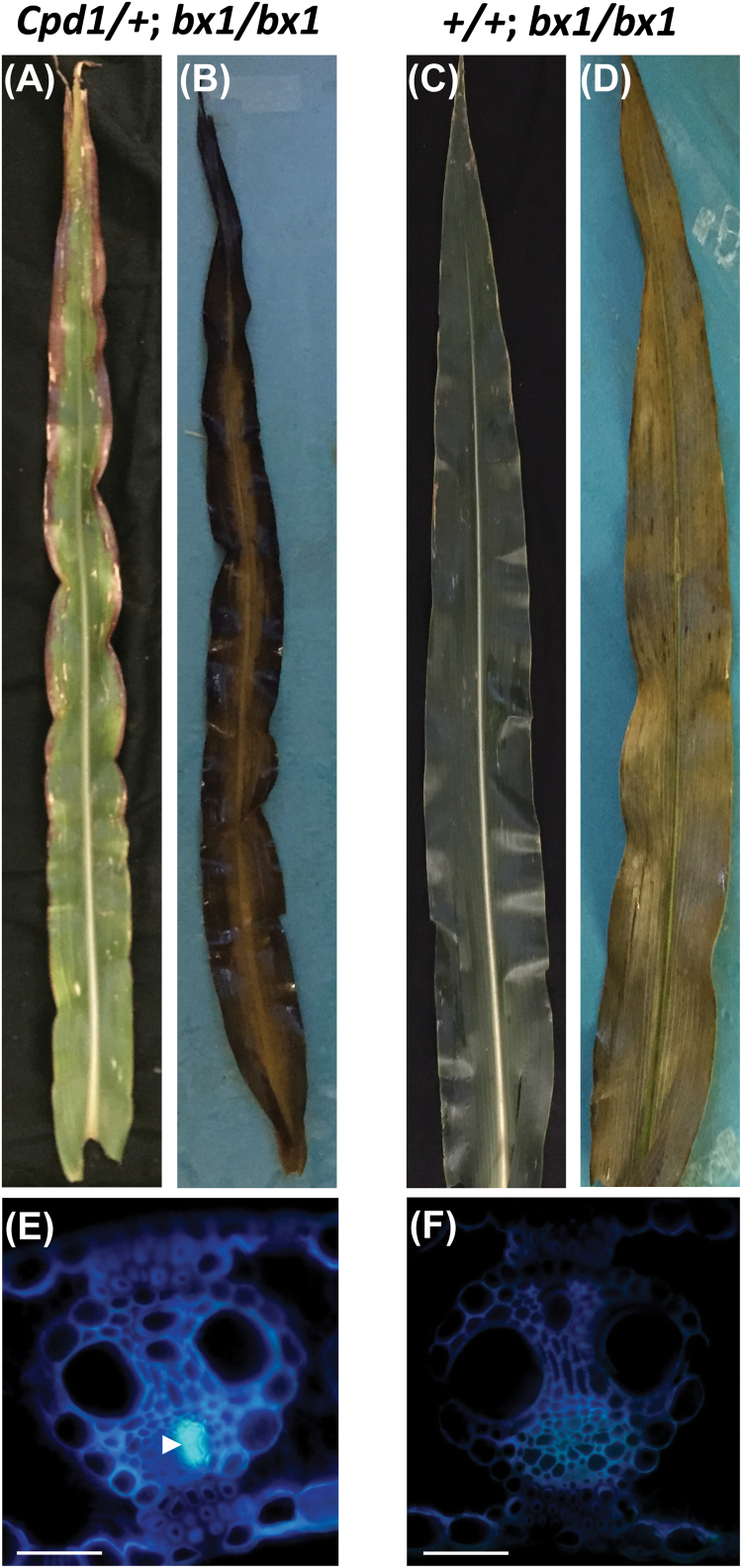
*Cpd1*/+; *bx1*/*bx1* double mutant plants exhibit the *Cpd1/+* mutant leaf phenotype of anthocyanin (A) and starch accumulation (B) relative to *+*/+; *bx1*/*bx1* (C and D) leaves. Additionally, *Cpd1*/+; *bx1*/*bx1* double mutants exhibit ectopic callose deposition in the phloem (arrowhead in E) compared with *+*/+; *bx1*/*bx1* controls (F). Scale bars=50 µm.

*Bx1* and *Bx2* encode enzymes that catalyze the first and second steps, respectively, in the synthesis of a variety of benzoxazinoid defense compounds, and *bx1/bx1* and *bx2/bx2* mutants lack benzoxazinoids (Frey *et al.*, 2009; Tzin *et al.*, 2017). As DIMBOA has been shown to trigger callose formation (Ahmad *et al.*, 2011; Meihls *et al.*, 2013), we addressed whether increased benzoxazinoids were the cause of the ectopic callose deposition observed in *Cpd1/*+ mutant individuals, by generating F_2_ families segregating *Cpd1/+* and *bx1/bx1* or *bx2/bx2* mutants. If benzoxazinoids are required to induce callose deposition in *Cpd1/+* mutants, it was expected that *Cpd1*/+; *bx1/bx1* and *Cpd1*/+; *bx2/bx2* double mutant plants would not synthesize DIMBOA, and would therefore have reduced callose accumulation in the phloem. If correct, we predicted that this would alleviate the block in phloem transport and rescue the *Cpd1/+* mutant phenotype. However, upon examination, both *Cpd1*/+; *bx1/bx1* and *Cpd1*/+; *bx2/bx2* double mutant plants exhibited the canonical *Cpd1/+* mutant phenotypes of anthocyanin and starch accumulation in mature leaves, as well as the ectopic callose deposition in the phloem (Fig. 10A, B, E; Supplementary Fig. S2). *bx1/bx1* and *bx2/bx2* single mutants did not show these phenotypes (Fig. 10C–F; Supplementary Fig. S2). In addition, innate DIMBOA and DIMBOA-Glc levels were measured in *Cpd1*/*+* leaf tissue free from biotic attack, and did not differ from wild-type samples (Fig. 9B). Therefore, while *Cpd1*/+ mutant individuals exhibit high levels of benzoxazinoids upon aphid feeding, we conclude that the ectopic callose deposition and resulting inhibited sucrose transport are independent of benzoxazinoid levels.

## Discussion

We identified the *Cpd1/+* mutant by its leaf chlorosis and anthocyanin accumulation, decreased plant stature, and delayed growth and development. This suite of visible phenotypes is shared with other previously studied carbohydrate partitioning-defective mutants, including *sxd1*, *sut1*, *tdy1*, *tdy2*, and the *psc* loci from maize, as well as some sucrose transporter mutants in Arabidopsis, tobacco (*Nicotiana tabacum*), tomato (*Solanum lycopersicum*), and potato (*Solanum tuberosum*) (Riesmeier *et al.*, 1994; Russin *et al.*, 1996; Bürkle *et al.*, 1998; Braun *et al.*, 2006; Hackel *et al.*, 2006; Baker and Braun, 2008; Srivastava *et al.*, 2008; Slewinski *et al.*, 2009; Slewinski and Braun, 2010*b*). These mutants all have defects in sucrose export and/or phloem transport in leaves. Therefore, we hypothesized that the *Cpd1* mutation impairs carbohydrate partitioning, potentially from an inability to export sucrose from its source leaves. The sugar and starch overaccumulation, decreased rates of photosynthesis, and reduced [^14^C]sucrose and CF transport in mutant leaves lend support to this hypothesis.

In further agreement with this hypothesis, ectopic callose and lignin deposition were found in some SEs in the phloem of *Cpd1* mutants. None of the other characterized maize carbohydrate hyperaccumulation mutants exhibit ectopic callose in their sieve tubes. Lignin deposits were found only in the distal tip regions of strongly expressing *Cpd1* mutant leaves, but not earlier in development, and do not seem to be the cause of the hyperaccumulation of carbohydrates. However, the observation of callose deposits in young, immature, and etiolated leaf tissue indicated that the callose deposition preceded the increased starch and soluble sugar accumulation and was independent of excess sugar or light signaling pathways. Additionally, we determined that the ectopic callose deposition in the phloem occurred very early during the development of mutant leaves (Figs 6, 7). However, in order for *Cpd1/+* mutant plants to survive, unoccluded SEs must also form normally to transport photosynthate throughout the plant. This suggests that the ectopic callose depositions observed in *Cpd1/+* mutant veins are not due to a systemic defect in phloem development, but instead must be dependent on a variable, local signal acting early in vein development.

Callose is a structural polymer in the cell wall that is formed through β-1,3 linkages of glucose molecules by callose synthases (Chen and Kim, 2009). It plays an important role in the formation of sieve pores located at the connecting ends of SEs (Barratt *et al.*, 2011; Xie *et al.*, 2011). The exact size and number of pores in the sieve plate directly impact the flow of phloem sap throughout the plant. For example, the Arabidopsis *Glucan Synthase7* (*gsl7*) callose synthase mutant results in the loss of callose lining the sieve plate pores, resulting in improperly formed sieve plates and a decreased ability to transport photosynthate to the flowering stem (Barratt *et al.*, 2011). Furthermore, this mutation also resulted in elevated levels of starch and anthocyanin accumulation in leaves compared with wild-type plants, a phenotype shared with the *Cpd1/+* mutant. However, in contrast to the *gsl7* mutant, the *Cpd1/+* mutation resulted in increased levels of callose deposition in the SEs, thereby limiting phloem transport of sucrose and presumably other molecules, such as hormones, RNAs, and proteins.

Intriguingly, there have been several mutants reported to show decreased export of photosynthate from the source leaves as a result of callose deposition in the vasculature. Callose deposition specifically at the BS cell–PP cell PDs was found in the maize *sxd1* mutant, a defect that probably resulted in decreased symplasmic transport and therefore hyperaccumulation of NSCs in the leaves (Russin *et al.*, 1996; Botha *et al.*, 2000; Provencher *et al.*, 2001). Similar phenotypes were found in terms of the leaf NSC accumulation and callose deposition in potato plants with reduced expression of the *sxd1* ortholog (Hofius *et al.*, 2004). Additionally, studies of the *sxd1* ortholog in Arabidopsis, *vitamin E1*, found similar NSC hyperaccumulation in mutant leaves, but only under low temperature conditions (Maeda *et al.*, 2006). It was determined that all three orthologous genes function in tocopherol synthesis (Sattler *et al.*, 2003; Hofius *et al.*, 2004; Maeda *et al.*, 2006). The reduced plant growth, NSC accumulation in leaves, ectopic callose deposition in veins, and reduced carbon exported from leaves of plants grown only at low temperatures was even more pronounced by a mutation in an upstream step of tocopherol biosynthesis encoded by the *VTE2* gene of Arabidopsis (Maeda *et al.*, 2006). However, in this case, callose deposition first occurred in a site-specific pattern in the phloem. Specifically, callose deposition was first observed at the transfer cell wall ingrowths at the boundary between the PP transfer cells and CC/SE complexes, and progressed to encircle the PP cells, including the PD connections with adjoining cells. Therefore, lack of tocopherols appears to induce callose deposition in the phloem tissue, specifically in PP cells, but not in the SEs. In contrast, in the *Cpd1/+* mutant, accumulation of ectopic callose occurred in the phloem SEs but was undetectable in the CCs and PP cells. However, the presence of callose in these two cell types cannot be precluded due to the limitations of resolution with light microscopy, and electron microscopy studies will be needed to resolve the cell type specificity of the ectopic callose. Nonetheless, due to the observable difference in cell-specific callose deposition, we do not favor the hypothesis that *Cpd1* is involved in tocopherol production.

We had initially hypothesized that the *Cpd1/+* mutation led to a constitutive defense response, which included ectopic callose plugging of the SEs, as has been shown to occur during pathogen attack, but subsequent findings led us to revise this idea. Interestingly, in citrus (*Poncirus trifoliata*) trees, citrus greening disease, which is caused by *Candidatus Liberibacter asiaticus* infection, results in callose deposition over the PD pore units between the CCs and SEs, as well as in the SEs themselves, blocking phloem transport (Koh *et al.*, 2012). A similar defense response to pathogens and hemipteran pests has been reported in maize, rice (*Oryza sativa*), tobacco, and Arabidopsis (Will and van Bel, 2006; Yun *et al.*, 2006; Hao *et al.*, 2008; Luna *et al.*, 2011; Tzin *et al.*, 2017). Ahmad *et al.* (2011) showed that the accumulation of benzoxazinoid compounds in the apoplasm of maize leaves results in increased callose deposition. While the cellular localization of the callose deposits was not determined, this theoretically could result in a blockage of sucrose transport if localized to the phloem. If so, this would effectively cut off the food supply and reduce infection by pests and pathogens attacking the plant. Therefore, we hypothesized that the excess callose deposition in the SEs of *Cpd1/+* mutant leaves could be due to increased benzoxazinoid levels.

Consistent with this idea, DIMBOA and its inactive form DIMBOA-Glc were present in 2- to 3-fold higher amounts in the *Cpd1/+* mutant after aphid feeding compared with wild-type plants. The results of the caterpillar and aphid feeding experiments further supported our initial hypothesis that a hyperactive defense response in the *Cpd1/+* mutant could result in its inability to transport phloem sap properly. Both of these results potentially contribute to the reduced fecundity of aphids feeding on the mutant leaves. Moreover, whereas beet armyworms and southern armyworms prefer to feed on various eudicot vegetable crops, fall armyworm feed primarily on monocots. The fall armyworm is not only more specialized for feeding on maize than the other two tested caterpillar species, but also has a specific detoxification mechanism for benzoxazinoids (which are induced more in *Cpd1/+* than in control plants by aphid feeding, Fig. 9) (Ali and Agrawal, 2012; Maag *et al.*, 2014). Since fall armyworms are relatively impervious to maize chemical defenses, this could explain why they grow better on *Cpd1/*+ mutant leaves, but the other two caterpillar species do not.

To test directly whether DIMBOA is required for the *Cpd1/+* mutant phenotype, double mutants with stocks devoid of benzoxazinoids were created. However, genetic tests of the causative role for benzoxazinoids in underlying the *Cpd1/+* mutant phenotype failed: *Cpd1/*+; *bx1/bx1* and *Cpd1/*+; *bx2/bx2* double mutant plants exhibited ectopic callose depositions similar to the *Cpd1/*+ single mutant individuals, demonstrating that the ectopic callose deposition observed in *Cpd1/+* mutant veins is independent of benzoxazinoid levels. Additionally, innate DIMBOA levels in *Cpd1*/+ mutant plants were similar to those of wild-type individuals. Based on these data, we do not favor our initial hypothesis presented above for *Cpd1* functioning to induce a hyperactive defense response, and further revise it to account for the *Cpd1/+*; *bx* double mutant studies such that it is independent of benzoxazinoid accumulation. However, a potentially more parsimonious hypothesis is that the increased tolerance to insect feeding and enhanced DIMBOA levels post-aphid treatment seen in *Cpd1/+* mutant plants is not due to a hyperactive defense response, but instead is due to priming of the defense response caused by an abundance of carbohydrates (Gebauer *et al.*, 2017). In this case, the *Cpd1/+* mutant plant would be able to synthesize an abundance of defense compounds quickly compared with wild-type siblings.

An alternative hypothesis that we favor to explain the *Cpd1/+* ectopic callose is that it results from a defect in sieve plate development during SE differentiation and maturation. During sieve plate development, callose is deposited around PDs in the end walls of the maturing SEs, signifying the location of future pores. Later, the callose surrounding the PD/pore is degraded up to the point where it lines the pore (Esau *et al.*, 1962; Northcote and Wooding, 1966). The proper formation and function of these pores ensure phloem sap transport through the sieve tubes at maturity (Barratt *et al.*, 2011). If this process is misregulated in the *Cpd1/+* mutant, it could result in either excessive deposition of callose or a failure to initiate callose degradation, with either scenario resulting in callose occlusion of the SEs. In support of this hypothesis, callose deposition was first observed very early during the development of the SEs in the lateral veins (Fig. 7) and to increase in severity over time.

As *Cpd1/+* is a semi-dominant mutation, it is interesting to speculate whether the *Cpd1* mutation is a gain- or loss-of-function mutation, and what the biological role of the wild-type *cpd1* gene may be. For example, it is possible that *Cpd1* is a gain-of-function mutation, and results either directly or indirectly in the constitutive deposition of callose in the SEs (Vatén *et al.*, 2011). On the other hand, *Cpd1* could be a haploinsufficient loss-of-function mutation, with the wild-type gene encoding a regulator of callose synthesis or an upstream component of this pathway. Under this scenario, insufficient *cpd1* gene product would be produced and not function to repress callose deposition, resulting in the occluded SEs. Further investigation is required to identify and characterize the function of the wild-type *cpd1* gene to understand how it controls phloem development and callose synthesis/degradation, and ultimately carbohydrate accumulation in leaves. Genetic fine-mapping and whole-genome sequencing approaches have delimited the region containing the *cpd1* locus to contain 10 predicted ORFs. None of these is predicted to have any biological functions in callose synthesis or degradation, tocopherol biosynthesis, PD development or function, production of defense metabolites, sucrose transport, starch metabolism, or vein development. Because *Cpd1* probably resulted from a single base pair change, is a semi-dominant mutation, and is represented by a single mutant allele, transgenic approaches to test these candidate genes have been initiated to identify the gene and causative mutation, but these experiments will take a considerable time. However, our characterization of *Cpd1* has uncovered a genetic lesion influencing callose accumulation in developing phloem SEs, and possibly the CCs and PP cells in maize leaves, which to our knowledge is a novel phenotype. We note that the ectopic callose occurs in only some phloem sieve tubes, suggesting that the mutation sensitizes some but not other developing phloem cells to deposit callose. We do not understand the factors underlying this deposition, but it does not appear to be related to plant age or size, growth environment (etiolated versus mature field grown leaves), phloem position with the vein, vein size, or vein length (lateral versus minor veins). However, it is more severe and frequent in the homozygous mutant individuals compared with the heterozygotes, consistent with the mutant allele conferring the phenotype in a dose-dependent manner. Understanding the function of *cpd1* will potentially have broad applications in unraveling the intersections among the induction of callose synthesis, sieve plate development, phloem function, plant defense priming, and carbohydrate partitioning, and it could provide knowledge valuable to generate crops with increased carbohydrate transport and yield.

## Supplementary data

Supplementary data are available at *JXB* online.

Fig. S1. Differences in photosynthetic capacity and gas exchange measurements between wild-type (WT) and chlorotic regions of *Cpd1*/+ mutant sibling leaves.

Fig. S2. *Cpd1*/+; *bx2*/*bx2* double mutant plants exhibit the *Cpd1/+* mutant leaf phenotype relative to *+*/+; *bx2*/*bx2* leaves.

Table S1. χ^2^ table for 1:1 segregation of *Cpd1*/+: wild-type families.

Supplementary Table S1

Supplementary Figures S1-S2

## Abbreviations:

BS: bundle sheath
bx1: *benzoxazinoneless1*
bx2: *benzoxazinoneless2*
CC: companion cell
CF: carboxyfluorescein
CFDA: carboxyfluorescein diacetate
Cpd1: *Carbohydrate partitioning defective1*
DIMBOA: 2,4-dihydroxy-7-methoxy-1,4-benzoxazin-3-one
DIMBOA-Glc: glucosylated-DIMBOA
EOD: end of day
EON: end of night
HPAE: high-performance anion exchange
M: mesophyll
NSC: non-structural carbohydrate
PD: plasmodesma
PP: phloem parenchyma
SE: sieve element
