# Supplementary material for: Maize Carbohydrate partitioning defective1 impacts carbohydrate distribution, callose accumulation, and phloem function
Source: J Exp Bot. 2018 May 26;69(16):3917–31. doi: 10.1093/jxb/ery203 (PMC6054164; doi:10.1093/jxb/ery203)
Supplement: Supplementary Table S1 [file ery203_suppl_supplementary_table-s1.pdf]

Supporting Information Table S1. χ^2 table for 1:1 segregation of *Cpd1*/+: wild-type families

Family No.	Observed *Cpd1*/+	Total Plants in Family	Expected *Cpd1*/+	p-value
1	2	6	3	
2	13	25	12.5	
3	10	22	11	
4	9	12	6	
5	5	14	7	
6	8	15	7.5	
7	11	19	9.5	
8	10	18	9	
9	6	14	7	
10	7	16	8	
Total	81	161	81.5	0.9574
