# Supplementary material for: Maize Carbohydrate partitioning defective1 impacts carbohydrate distribution, callose accumulation, and phloem function
Source: J Exp Bot. 2018 May 26;69(16):3917–31. doi: 10.1093/jxb/ery203 (PMC6054164; doi:10.1093/jxb/ery203)
Supplement: Supplementary Figures S1-S2 [file ery203_suppl_supplementary_figures-s1-s2.pdf]

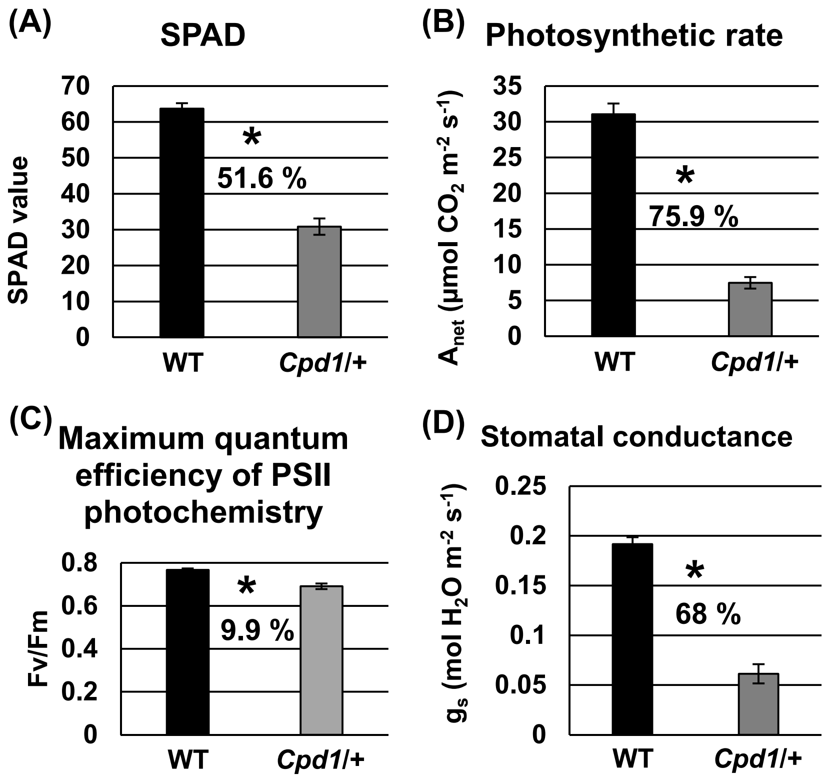

Supplemental Figure S1. Differences in photosynthetic capacity and gas exchange measurements between wild-type (WT) and chlorotic regions of *Cpd1*/+ mutant sibling leaves. (A) Relative chlorophyll content. (B) Leaf net assimilation rate (A_{net}). (C) Maximum quantum efficiency of photosystem II (PSII) (F_v/F_m). (D) Stomatal conductance (g_s). Values are means ± SE, and all means between WT and *Cpd1*/+ mutants are significantly different at $p \leq 0.05$, using a two-tailed Student's *t*-test. Percentages indicated represent the differences between the two means.

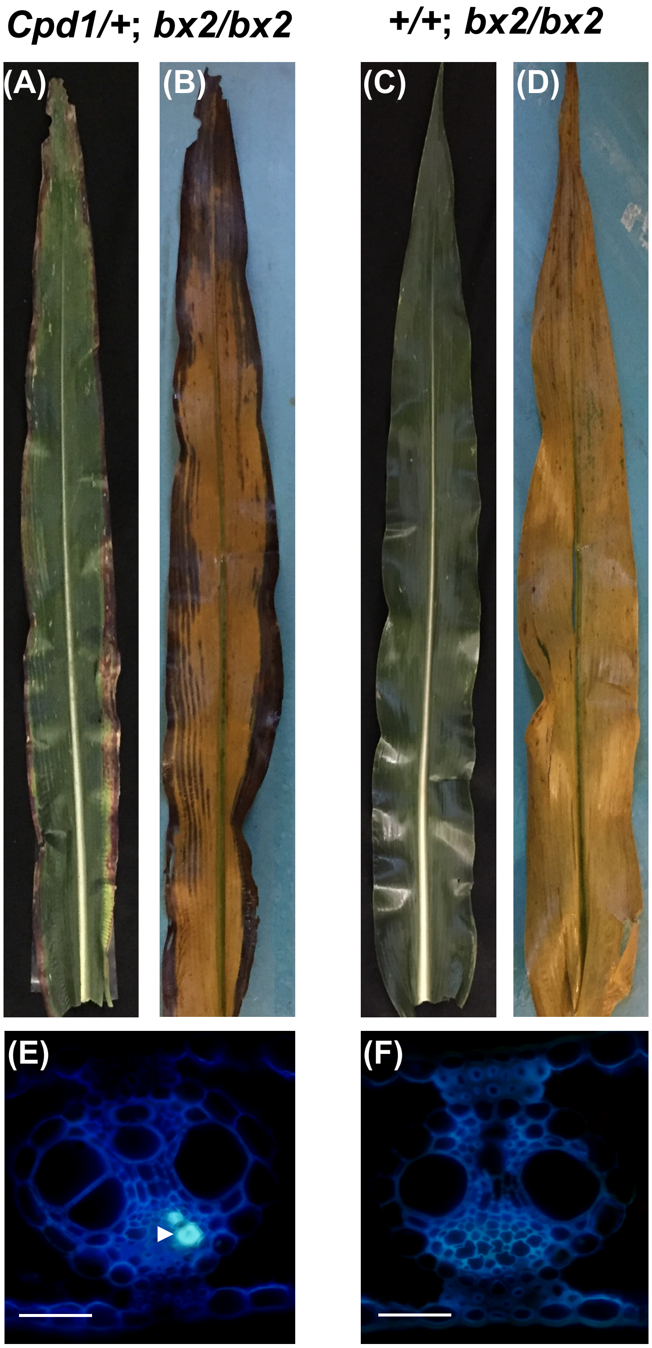

Supporting Information Figure S2. *Cpd1*/+; *bx2*/*bx2* double mutant plants exhibit the *Cpd1/*+ mutant leaf phenotype of anthocyanin (A) and starch accumulation (B) relative to +/+; *bx2*/*bx2* leaves (C-D). Additionally, *Cpd1*/+; *bx2*/*bx2* double mutants exhibit ectopic callose deposition in the phloem (arrowhead in E) compared to +/+; *bx2*/*bx2* controls (F). Scale bars = 50µm
